# Pan-Cancer Analysis of Radiotherapy Benefits and Immune Infiltration in Multiple Human Cancers

**DOI:** 10.3390/cancers12040957

**Published:** 2020-04-13

**Authors:** Pengbo Wen, Yang Gao, Bin Chen, Xiaojing Qi, Guanshuo Hu, An Xu, Junfeng Xia, Lijun Wu, Huayi Lu, Guoping Zhao

**Affiliations:** 1 Key Laboratory of High Magnetic Field and Ion Beam Physical Biology, Hefei Institutes of Physical Science, Chinese Academy of Sciences; Anhui Province Key Laboratory of Environmental Toxicology and Pollution Control Technology, Hefei 230031, China; wenpb@mail.ustc.edu.cn (P.W.); gy.sunny@foxmail.com (Y.G.); upets@mail.ustc.edu.cn (B.C.); qxj0308@mail.ustc.edu.cn (X.Q.); hgs12345@mail.ustc.edu.cn (G.H.); anxu@ipp.ac.cn (A.X.); ljw@ipp.ac.cn (L.W.); 2 University of Science and Technology of China, Hefei 230026, China; 3 Institute of Physical Science and Information Technology, School of Computer Science and Technology, Anhui University, Hefei 230039, China; jfxia@ahu.edu.cn; 4 Department of Ophthalmology & Visual Sciences, Division of Life Sciences and Medicine, University of Science and Technology of China, Hefei 230026, China

**Keywords:** pan-cancer, radiotherapy, immune infiltration, prognosis

## Abstract

Response to radiotherapy (RT) in cancers varies widely among patients. Therefore, it is very important to predict who will benefit from RT before clinical treatment. Consideration of the immune tumor microenvironment (TME) could provide novel insight into tumor treatment options. In this study, we investigated the link between immune infiltration status and clinical RT outcome in order to identify certain leukocyte subsets that could potentially influence the clinical RT benefit across cancers. By integrally analyzing the TCGA data across seven cancers, we identified complex associations between immune infiltration and patients RT outcomes. Besides, immune cells showed large differences in their populations in various cancers, and the most abundant cells were resting memory CD4 T cells. Additionally, the proportion of activated CD4 memory T cells and activated mast cells, albeit at low number, were closely related to RT overall survival in multiple cancers. Furthermore, a prognostic model for RT outcomes was established with good performance based on the immune infiltration status. Summarized, immune infiltration was found to be of significant clinical relevance to RT outcomes. These findings may help to shed light on the impact of tumor-associated immune cell infiltration on cancer RT outcomes, and identify biomarkers and therapeutic targets.

## 1. Introduction

Radiotherapy (RT) is the primary method for cancer treatment given to approximately 60% of all newly diagnosed patients [1]. Significant physical advances in RT have been achieved by developing methods of treatment planning and delivery [2]. However, due to differences in tumor radiosensitivity, not all patients derive survival benefit from RT, while suffering serious adverse consequences [3,4]. Thus, radiosensitivity prediction has always been a topic of primary importance in the field of biologically guided personalized treatment strategies in radiation oncology [5,6].

In the current era of precision medicine, high-throughput technologies have provided an opportunity to approach the development of radiosensitivity biomarkers from a different perspective. For example, based on a 10-gene signature, Eschrich et al. developed the radiosensitivity index (RSI), which is directly proportional to tumor radioresistance [7]. Further, Speers et al. created a human breast cancer (BRCA)-specific radiosensitivity signature (RSS) with biological relevance and validated this signature for the prediction of local recurrence [8]. Finally, Gene Ontology (GO) analyses were employed to define key molecular biomarkers governing response to radiation in rectal cancer [9]. However, all these studies were based on the intrinsic radiation response of tumor cells and the effects of the TME on this response were ignored [10,11].

For decades, research into improving outcomes from RT focused almost entirely on the cancer cell itself, ignoring complex biological interactions between the tumor and the stroma in which it grows—the so-called TME. As a result, classical radiobiology largely failed to appreciate that the effects of RT on the TME, and the responses that are triggered within it, may be critical in determining the success or failure of therapy. TME is the environment around a tumor, composed of a dynamic, disorganized, and corrupted mixture of a variety of different molecules [12,13]. TME were reported to closely associate with tumor growth, metastasis, and response to clinical treatment [1,14,15,16,17]. Due to its negative effect on the TME, radiation itself was considered to be immunosuppressive. Recently, TME was regarded as a “Game Changer” in designing RT [18]. On one hand, the effect of radiation on tumor cells could induce the release of new antigens and trigger the immune system to activate tumor-specific T cells. On the other hand, radiation could enhance TME immune infiltration, thereby overcoming some of the barriers to tumor rejection [19,20,21]. Cancer cells often express programmed cell death-ligand 1 (PD-L1) and subsequently induce T-cell apoptosis, and PD-L1 status is an important factor in the prediction of the clinical outcome following RT in BRCA patients [22]. However, a comprehensive analysis of the correlation of RT outcome with immune infiltration has not yet been reported [23].

To comprehensively evaluate clinically relevant immune infiltration in RT, which may function as a prognostic predictor of different cancer types, we integrated and analyzed clinical information and RNA-sequencing data from seven cancers in this study. In addition, to demonstrate the potential clinical translational value of our findings, an immune infiltration-based prognostic signature was developed, which represents a promising tool for overall survival (OS) prediction in BRCA patients with RT treatment.

## 2. Results

### 2.1. Subsection

#### 2.1.1. Patient and Tumor Characteristics in Seven Human Cancers

**Figure 1:**
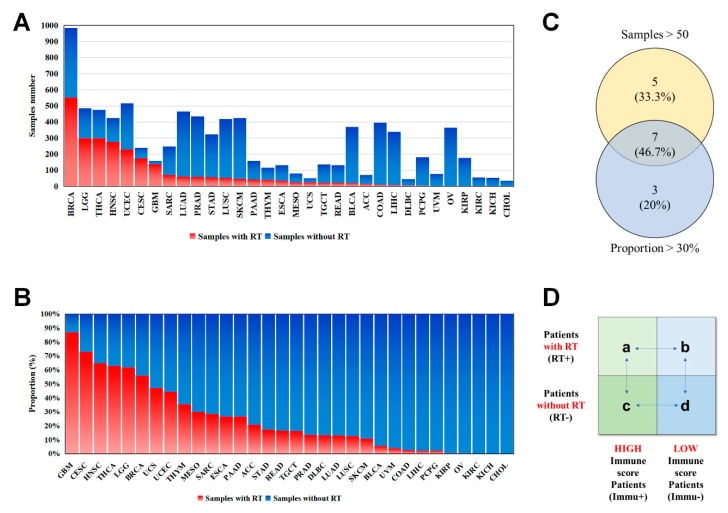
Overview of the samples downloaded from TCGA. (**A**) Number of samples for each cancer type were shown, sorted by the number of samples receiving radiation therapy. (**B**) The percent adoption of RT in different cancers. The red and green represent the patients with or without RT, respectively. (**C**) Cancers with a total sample size greater than 50 and a RT application rate greater than 30% were selected for subsequent analysis. (**D**) According to the immune infiltration level and RT status, the patients with each cancer were divided into four different subgroups. Immune Score was calculated by ESTIMATE algorithm. Take subgroup (**a**) as an example, these patients received RT and their tumors had a high level of immune infiltration. In the subsequent analysis, we compared the survival differences between different subgroups, they are: (**a** + **b**) vs. (**c** + **d**), (**a**) vs. (**b**), (**a**) vs. (**c**), (**b**) vs. (**d**), (**c**) vs. (**d**), respectively.

Integrated analysis was performed on patients in The Cancer Genome Atlas (TCGA) cohort (Supplementary Table S1). For different cancer types, the number of samples (with RT information) ranged from 984 in the case of BRCA to 35 in the case of CHOL (Figure 1A). Sample sizes greater than 50 were considered in our analysis, including breast invasive carcinoma (BRCA, 551 samples), brain low-grade glioma (LGG, 299 samples), thyroid carcinoma (THCA, 299 samples), head and neck squamous-cell carcinoma (HNSC, 275 samples), uterine corpus endometrial carcinoma (UCEC, 229 samples), cervical squamous-cell carcinoma and endocervical adenocarcinoma (CESC, 175), and glioblastoma multiforme (GBM, 138). The percent adoption of RT in different cancers ranged from 86.79% to 0.00% (Figure 1B). The 10 cancers in which more than 30% of patients received radiation therapy, in order of RT treatment frequency, were: GBM (86.79%), CESC (72.92%), HNSC (64.86%), THCA (62.95%), LGG (61.78%), BRCA (56.00%), uterine carcinosarcoma (UCS; 47.06%), UCEC (44.38%), thyroid carcinoma (THYM; 35.34%), and mesothelioma (MESO; 30.00%) (Figure 1B). After comprehensive consideration, seven types of cancers were selected for subsequent analysis (Figure 1C). The characteristics of patients with one of the seven types of cancers are summarized in Supplementary Table S2.

#### 2.1.2. Profiles of Immune Infiltration and RT Outcome

The correlation between immune infiltration and cancer stage is widely recognized. Currently, the cancer stage is an important indicator for RT management. However, we found that the cancer stage is not a universal indicator for the RT management by univariate cox analysis (Table 1). Thus, we hold the opinion that the immune infiltration level is a powerful supplement for the existing clinical indicators.

**Figure 2:**
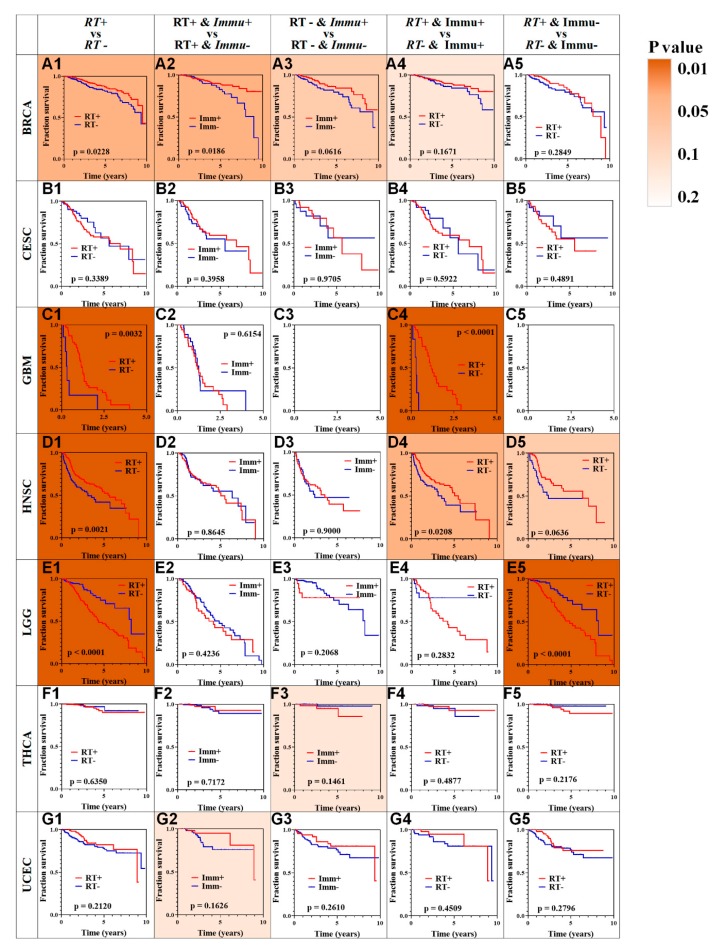
Survival analysis across seven cancer types. Each row represents a type of cancer and each column represents a different grouping. For each type of cancer (**A**–**G**), we performed five different survival analysis (numbers 1–5, refer to Figure 1D). A Kaplan–Meier Plotter was used to test for survival prediction capacity. A combination of letters (**A**–**G**) and numbers (1–5) were used to number all the results. Correspondingly, the patients with an immune score greater than zero were defined as positive (+), and those with a score below zero were defined as negative (-). Additionally, patients with or without RT treatment were defined as positive (+) or negative (-), respectively. A total of seven types of cancer were analyzed: BRCA (**A1**–**A5**), CESC (**B1**–**B5**), GBM (**C1**–**C5**), HNSC (**D1**–**D5**), LGG (**E1**–**E5**), THCA (**F1**–**F5**), UCEC (**G1**–**G5**). For C3 and C5, the data was insufficient for analysis. The fill color was related to the *p* value, the darker the more statistically significant.

To verify the relationship between immune infiltration and RT outcome for each cancer, whole samples were classified into four different subgroups (Figure 1D) according to each sample’s immune infiltration and RT status. More specifically, after calculating the immune infiltration level of each patient using the ESTIMATE algorithm, we divided the patients into positive and negative groups. The patients with an immune score greater than zero were defined as positive, and those with a score below zero were defined as negative. Then, we compared the prognosis of each patient subjected to RT. Pan-cancer survival analysis showed that not all cancer types benefit from RT (Figure 2, first column of Figure 2).

To evaluate the relationship between immune infiltration and patient prognosis, our analysis also included those patients without receiving RT and made all the possible subsets (second-fourth column of Figure 2). According to our analysis, for BRCA patients (Figure 2A1), RT had significant positive association with OS (Figure 2A1, *p* = 0.0228). Besides, higher level immune infiltration improved BRCA patients’ OS (*p* = 0.0186; Figure 2A2). For LGG patients with negative immune status, RT could greatly improve OS (*p* = 0.0001; Figure 2E5). For HNSC and GBM patients with positive immune status, RT could also greatly improve OS time (*p* = 0.0208 and *p* = 0.0001, respectively (Figure 2D4, Figure 2C4). By contrast, RT and immune infiltration had no effect on patients with THCA, UCEC, or CESC (Figure 2F, Figure 2G, Figure 2D). Overall, immune infiltration levels were associated with patients’ RT outcomes, which have considerable significance in guiding decisions in the clinical context.

#### 2.1.3. Immune Cell Subpopulations and RT Outcomes

The level of immune infiltration is determined by the number of immune cell types in the TME. Depending on cell type and functional interactions, immune cells play a central role in resisting or accelerating tumor growth in patients through their behaviors, such as defending against, or obliterating, potential hazards. Accordingly, in this section, we intended to find immune cells that are related to the prognosis of patients receiving radiotherapy.

**Figure 3:**
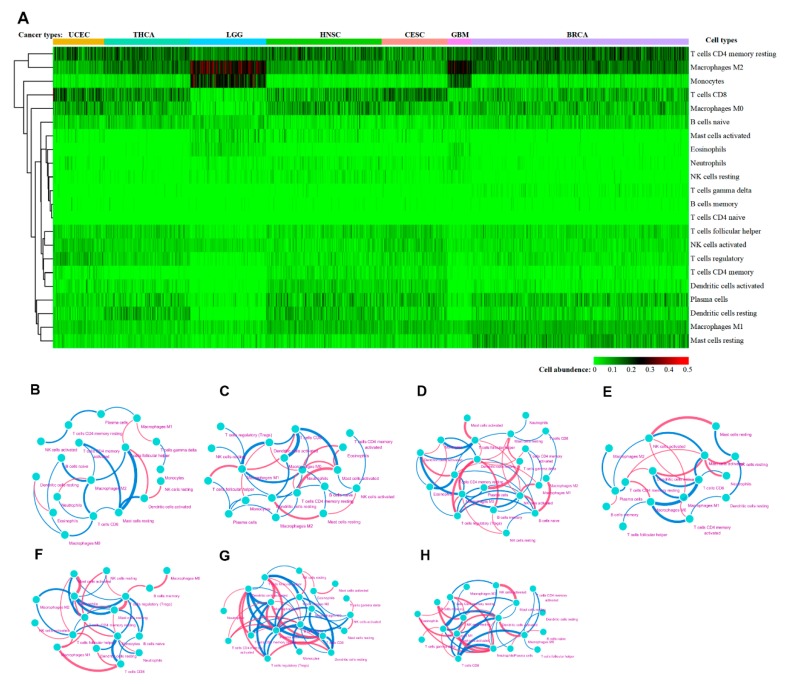
Composition of immune cells and their correlations across seven cancer types. (**A**) Heatmap was used to exhibit the different immune cells abundance across seven cancer types. Each column represents a patient sample, and each row represents an immune cell. The color represents the abundance of the different immune cells. The color bars above the picture represent different types of cancer, they are UCEC, THCA, LGG, HNSC, CESC, GBM, BRCA, respectively. (**B**–**H**) Correlation of 22 immune cells in TME across seven cancer types, among which the line thicknesses and color represented correlation, blue represented negative correlation, red represented positive correlation, and the thicker the line, the larger the correlation coefficient. Each dot in the figure represents an immune cell. In these figures, only the cells with high correlation coefficients were shown.

Owing to technical limitations, accurate information about immune cell distribution in TME cannot be easily acquired. Here, to explore the relationship between immune cell composition in the TME and prognosis of RT, CIBERSORT algorithm was used to characterize leukocyte subsets for each patient from the gene expression profiles. Based on unsupervised hierarchical clustering, the heat map shows levels of immune cell composition for the seven types of cancer patients (Figure 3A). It indicated that immune cell composition was markedly distinct among different cancer types. The levels of resting CD4 memory T cells, M2 macrophages, and CD8 T cells were high in many cancer types, while gammadelta T cells, naive CD4 T cells and memory B cells constituted a low proportion of the total cells. Furthermore, glioma tissue contained the highest percentages of M2 macrophages and monocytes among all cancers. To explore the potential connections of immune cells, the correlation coefficients between 22 immune cells based on their abundance were calculated (Supplementary Figure S1–S7). Accordingly, the correlation coefficients in the form of a network were visualized (Figure 3B–H). Although, the correlation coefficient cannot be quantitatively compared between different cancers, due to the difference in the sample size of each cancer. It could illustrate that the association of immune cells in each type of cancer varies widely.

**Figure 4:**
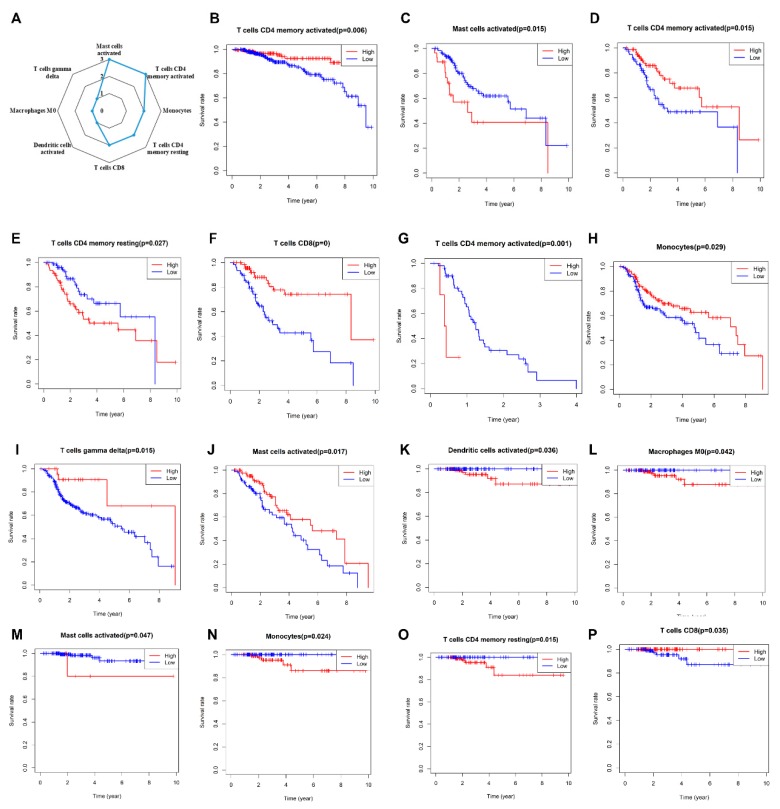
Prognostic value of 22 tumors infiltrating immune cells across seven cancer types. The radar chart (**A**) gives an overview of the cell types with prognostic value. According to the Kaplan–Meier analysis, only cells that displayed significant correlation RT outcomes were shown in this figure (**B**–**P**). Red lines indicate high immune infiltration of a certain cell, and blue lines indicate low immune infiltration. Each picture corresponds to a different type of cancer BRCA (**B**), CESC (**C**–**F**), GBM (**G**), HNSC (**H**,**I**), LGG (**J**), THCA (**K**–**P**).

Next, we inferred that divergence in immune cell subpopulation levels might serve as an essential proxy for individual differences and, therefore, hold prognostic value. Kaplan–Meier analysis was utilized to investigate the RT prognostic value of 22 tumors infiltrating immune cells across seven cancer types. According to the 154 (22 × 7) results of Kaplan-Meier analysis, we found that eight types of immune cells were associated with RT outcomes (Figure 4A). Among them, ‘T cells CD4 memory activated’ and ‘Mast cells activated’ were related to the RT outcomes in multiple cancer types (Figure 4A). Interestingly, as shown in Figure 4, a certain immune cell subset may have opposite effects on RT outcome in different types of cancer. For example, ‘mast cells activated’ was positively related to the 10-year OS of patients with LGG (Figure 4J), but negatively related to the patients with CESC (Figure 4C) and THCA (Figure 4M). A similar phenomenon was also found for ‘T cells CD4 memory activated’, high infiltration of ‘T cells CD4 memory activated’ was positively related with RT outcome in BRCA (*p* = 0.006) and CESC (*p* = 0.015), but negatively related in GBM (*p* = 0.001). Taken together, these findings suggest that immune cell subpopulations could provide additional prognostic value for RT outcomes.

#### 2.1.4. BRCA Radiosensitivity Signature Based on Immune Infiltration

**Figure 5:**
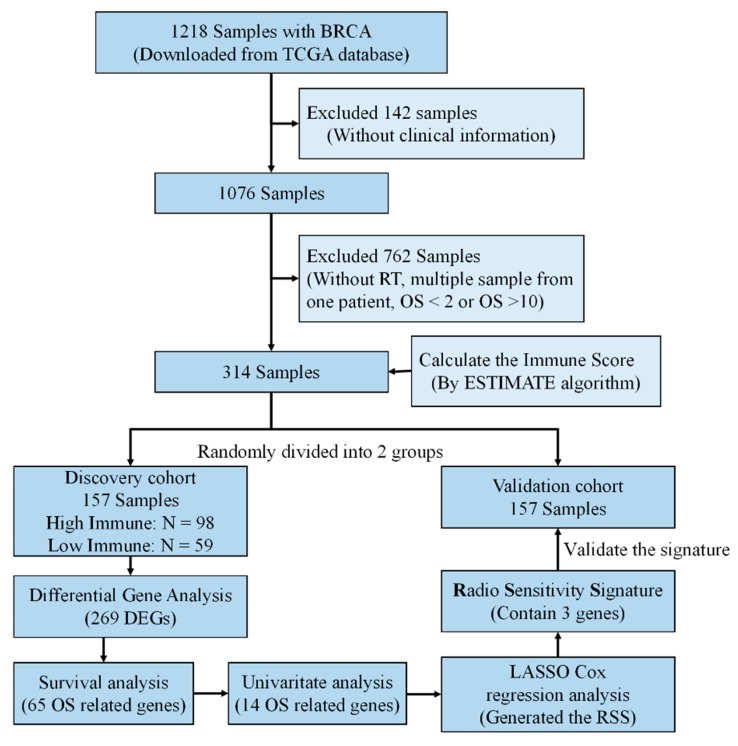
Study designs. A prognostic classifier was constructed in the discovery cohort (*n* = 157) and was further certified in the validation cohort (*n* = 157).

Previous studies have generated an RSS base on the intrinsic radiosensitivity in BRCA by the integration of post-radiation clonogenic survival data with gene expression data [11,24,25,26]. To demonstrate whether immune infiltration level has predictive ability for RT outcomes, we developed a Radiosensitivity Signature (RSS) in BRCA (Figure 5).

**Figure 6:**
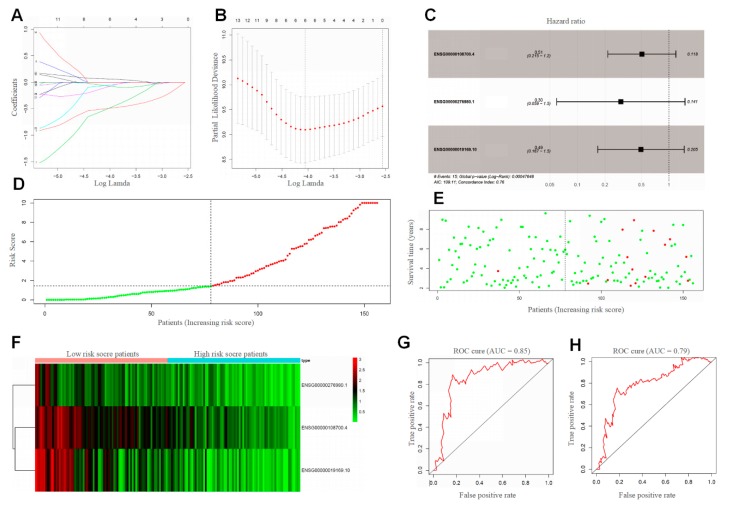
Construction and validation of the RSS. (**A**,**B**) Fourteen survival-related genes selected by LASSO Cox regression analysis. LASSO coefficient profiles of the immune-infiltrating cell. Right: using 10-fold cross-validation to the optimal penalty parameter lambda. All the genes’ HR <1 (**C**), which indicated that the variable decreases the likelihood of the outcome. (**D**,**E**) Distribution of the RSS in the discovery cohort. Left panel: classification of patients into different risk groups based on the optimal RSS. Right panel: distribution of patients’ survival time and status. (**F**) Heatmap for comparison between high risk score and low risk score patients for the expression of 3 genes. ROC analysis showed the diagnostic value of risk score in discovery cohort (**G**) and validation cohort (**H**).

Considering the clinical characteristics of BRCA (Supplementary Materials), we selected 314 samples from the TCGA dataset. These samples were equally divided into discovery and validation cohorts, respectively. Of the 268 curated differentially expressed genes (DEGs), 65 were significantly associated with RT patient outcomes (Supplemental Figure S8). The survival-related genes were further optimized by univariate regression analysis (Figure 5). To obtain the optimal cutoff values of the survival-related genes, Least Absolute Shrinkage and Selection Operator (LASSO) Cox regression analysis [27] was performed based on the survival associated with 14 genes in the discovery cohort (Figure 6A,B). Three-genes signature was curated to construct the prediction model (Figure 6C–F, Supplemental Table S3). The area under the receiver operating characteristic (ROC) curve for the immune infiltrating score was 0.853 (Figure 6G). Moreover, the risk score was an independent prognostic factor in the validation cohort, based on the univariate and multivariate Cox regression model (Supplemental Table S4). This indicated that the RSS has a good performance on discovery cohort.

To further validate the robustness of the 3-genes signature (the RSS), the validation group was applied to evaluate the prognostic value of the proposed scoring model. The same formulae for the immune cell infiltration score and the optimal cut-off point for each immune cell type were applied to the validation group as to the discovery group. Likewise, ROC analysis was also performed on the validation cohort to assess the prognostic value of the scoring model. The area under the curve was 0.79 (Figure 6H), which indicated the RSS has a high predictive ability. This was a good proof of the validity of the IRS model we constructed. In agreement with the results above, the effective prediction model further demonstrates that immune infiltration is a considerable indicator of clinical RT management and radiotherapy outcome precision.

## 3. Discussion

In this study, we performed an integrated analysis of immune infiltration and RT outcomes across multiple cancers. Based on the seven cancer types from the TCGA dataset, we found that immune invasion levels in certain types of cancers correlate with RT patient prognosis and that knowledge of immune cell subpopulation could provide additional prognostic value in the context of RT treatment. To our knowledge, our study firstly demonstrated the relationship between clinical RT outcomes and immune infiltration status in multiple cancer types. Additionally, we validated the immune infiltration-dependent RSS, which was alleged to reflect radiosensitivity in the BRCA dataset, and the result suggested that this gene signature could be a predictive marker for the response of these patients to RT.

As an example of a local ablative physical therapy, RT uses high energy radiation for local cancer treatment. It can induce double-strand DNA damage, single-strand breaks, misrepair, and chromosome aberrations in cancer cells [28]. As the most effective cytotoxic method available for the treatment of patients with solid tumors, RT has a wide range of applications [29]. Consistent with our statistics, RT is applied in over 60% of GBM, CESC, and HNSC cases. Besides, increasing survival time after treatment in patients with BRCA, LGG, or GBM, RT could also be used as palliative therapy to relieve symptoms and improve the quality of life of patients with CESC or UCEC.

For many years, in order to improve the outcomes of RT, most studies focused on mechanisms internal to the cancer cell (intrinsic radiosensitivity), ignoring complex interactions between the tumor and the TME in which it grows (extrinsic radiosensitivity) [19,20,21]. Moreover, pre-clinical studies in some tumor models have suggested that RT-induced changes in the TME might, in fact, promote tumor invasion and spread the tumor in certain situations [30,31,32]. Thus, attempts to combine RT with recent targeted therapies were often predicated on their potential to enhance radiation-induced cancer cell death rather than on their capacity to modify the interactions between the cancer cell and its surroundings.

Recently, researchers have developed the view that the complex reactions of the immune system to an irradiated TME are dual-purpose, performing both immunostimulatory and immunosuppressive functions [12,33]. Tumor-infiltrating immune cells play an indispensable role in such interactions. These cells migrate from the periphery to tumor tissues and exert vital functional roles in promoting and/or regulating tumor progression and growth. Specifically, radiation could exert effects on cells intrinsic to the TME, such as altered production of inflammatory cytokines, antigen exposure and DC priming, as well as relative increases in radioresistant immunosuppressive macrophage and T-cell populations. Furthermore, emerging evidence suggests that infiltrating immune cells play major roles in the diagnosis and treatment of cancer patients [33,34]. Hence, in this study, we focused on the prognostic value of immune infiltration to predict patient RT outcomes.

For the seven cancer types analyzed, we found that different types of infiltrating immune cells vary—not only among different types of cancers, but also within the same type of tumor, or at different time points in the same patient. Differences in immune cell composition may reflect tumor heterogeneity, but its relationship with tumor-infiltrating immune cells is controversial, with some authors proposing that tumors with high heterogeneity may generate neoantigens that attract immune cells and others claiming that immune cells provide selection pressure that shapes tumor heterogeneity [35,36,37].

In the upcoming era of combination immunotherapy, it is becoming critical to understand the TME immune infiltration in order to boost antitumor immunity. According to our previous research, it was found that most studies in recent decades focused only on the intrinsic radiation sensitivity in tumor cells [38], and ignoring complex biological interactions between the tumor and the stroma in which it grows [39]. In this study, we identified activated mast cells and the CD4 memory T cell that are closely related to patient RT outcomes. Mast cells are a part of the immune system, and it has been reported that the cell will accumulate in tissues undergoing angiogenesis during tumor growth, wound healing, and tissue repair. Mast cells can secrete angiogenic factors such as vascular endothelial growth factor (VEGF). Heissig et al. observed that irradiation fosters mast cell-dependent vascular regeneration in a limb ischemia model. They demonstrated that irradiation could promote VEGF production by mast cells in a matrix metalloproteinase-9 (MMP-9)-dependent manner [40]. Interestingly, in CESC and THCA (Figure 4C,M), activated mast cells were negatively correlated to RT outcome. Here, we hold the opinion that radiation could induce mast cell activation and VEGF releasing. Thus, the VEGF would promote the revascularization in the TME, cause tumor recurrence, and lead to poor RT outcome. Moreover, T cells are a type of white blood cell at the core of adaptive immunity, and tailors the body’s immune response to specific pathogens. According to previous studies, T-cell activation requires several signals: antigen in an appropriate major histocompatibility complex (MHC) binding to a T-cell receptor (TCR); co-stimulatory signals (e.g., from an APC); background levels of cytokine stimulation [41]. Radiation-induced neoantigens promote T-cell activation, thereby enhancing immune effects. Besides, low and high doses of radiation could have different effects on T cell activation [42]. It may explain our results, among which CD4 memory T cells are positively correlated with RT outcomes in BRCA and CESE, and negatively correlated in GBM. Although our study confirmed that immune cell abundance is closely related to RT outcome, the relationship between immune cell localization and RT outcomes need to be further studied.

Additionally, response to RT varies widely among cancer patients. Therefore, it is of substantial clinical importance to be able to predict which patients will benefit from RT before treatment is initiated. Prognostic signatures constructed by intrinsic tumor radiosensitivity have been reported in many studies. In this work, we developed an RSS based solely on the immune infiltration status (external radiosensitivity) of BRCA, and this RSS possesses a similar predictive power to other methods.

## 4. Materials and Methods

### 4.1. Data Acquisition

The expression data and corresponding clinical information of the patients were downloaded from TCGA (https://portal.gdc.cancer.gov/).

### 4.2. Inferring Tumor Purity

ESTIMATE [43] (Estimation of STromal and Immune cells in MAlignant Tumor tissues using Expression data) is an algorithm that uses gene expression signatures to infer the fraction of stromal and immune cells in tumor samples. The algorithm, stromal and immune scores can be calculated to predict the level of infiltrating stromal and immune cells in tumor tissue. More information can be acquired online at https://bioinformatics.mdanderson.org/public-software/estimate/. In this study, the ESTIMATE method was applied for assessment of the infiltration of immune cells in tumor samples using gene expression data that were downloaded from TCGA database. According to the immune score, all the samples were divided into high and low groups separately.

### 4.3. CIBERSORT Deconvolution Algorithm

To characterize the abundance of immune cells in malignant tissues, the CIBERSORT method [44] was adopted in our study. CIBERSORT is a computational approach that accurately resolves relative fractions of diverse cell subsets in GEPs from complex tissues. Basically, CIBERSORT requires an input matrix of reference gene expression signatures, collectively used to estimate the relative proportions of each cell type of interest [33]. We downloaded the leukocyte gene signature matrix, termed LM22 (http://cibersort.stanford.edu), which contains 547 genes that distinguish 22 human hematopoietic cell phenotypes, including seven T-cell types, naive and memory B cells, plasma cells, natural killer (NK) cells, and myeloid subsets. In combination with the LM22 signature matrix, CIBERSORT was used to estimate the fractions of 22 immune cell types in our study. As CIBERSORT computes an empirical *p*-value of deconvolution to denote the accuracy of results, we only retained the samples with CIBERSORT *p*-values < 0.05 for subsequent analysis. In the final output of CIBERSORT, the sum of the predicted immune cell type fractions was 1 within each sample, and thus, the outputs were directly integrated to generate an entire matrix of immune cell fractions.

### 4.4. BRCA Radiosensitivity Signature Construction and Validation

The patients were randomly separated into a discovery cohort and validation cohort. The discovery cohort was used to discover potentially predictive relationships. The validation set was used to validate the predictive power of the model that generated from discovery cohort. In the discovery cohort, after performing differentially expressed gene analysis, we used univariate Cox regression analysis to identify prognostic genes, and genes with a cut-off of *p*  <  0.05 were considered significant. Since too many genes are correlated with RT outcome, lasso-penalized Cox regression analysis was used to exclude genes with low correlation and to filter out highly related genes (ref). Then, a prognostic gene signature was constructed based on the linear combination of the regression coefficient derived from the lasso Cox regression model coefficients (β) multiplied with its mRNA expression level. The risk score  =  (β1 * Gene1)  +  (β2 * Gene2)  +  (β3 * Gene3) + ⋯ + (βN * GeneN). The optimal cut-off value was investigated by The R package “survival” and “survminer” and two-sided log-rank test. Patients were classified into a high-risk and low-risk cohort according to the threshold. The time-dependent receiver operating characteristic (ROC) curve was drawn to evaluate the predictive value of the prognostic gene signature for overall survival using the R package “survivalROC”.

### 4.5. Statistics and Analysis

All statistical tests were two-sided, and *p*-values of less than 0.050 were considered statistically significant. These tests were performed by R version 3.6.0 (http://www.r-project.org). Several R packages were used in our study including: “limma”, “e1071”, “parallel”, “survival”, “ggplot2”, “pheatmap”, “corrplot”, “glmnet”, “survminer”, “survivalROC”, “Hmisc”, “lattice”, “Formula”, “foreign”, “rms”. Additionally, the networks were visualized by Cytoscape 3.5.1 (https://cytoscape.org/).

## 5. Conclusions

**Figure 7:**
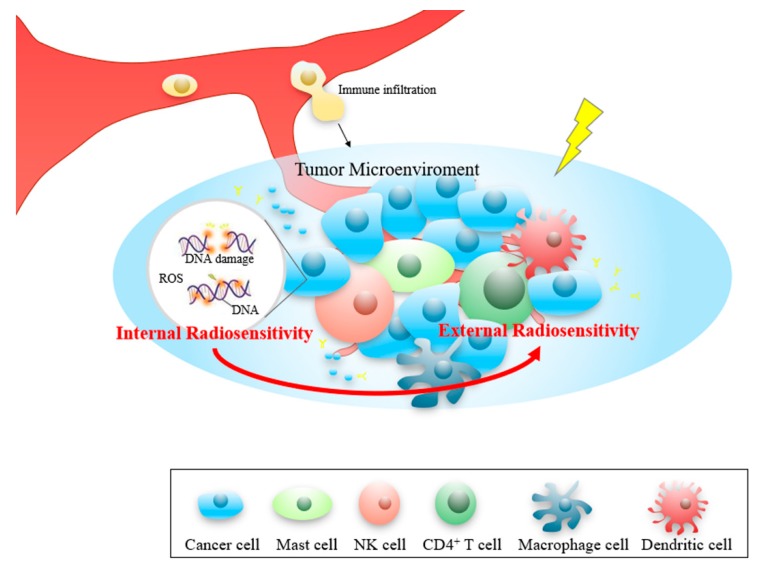
Overview of the study. Our research has initially demonstrated that not only the tumor cells intrinsic radiosensitivity, but the extrinsic radiosensitivity could also affect patients’ RT outcome.

In conclusion, our study performed a pan-cancer analysis to reveal that immune infiltration influences RT outcomes. Its findings have the potential to redirect focus in the field from intrinsic radiosensitivity to extrinsic radiosensitivity (Figure 7). Thus, it is imperative to explore the heterogeneity of immune cell indicators for prognostic prediction in multiple cancers in the near-term and potentially for individualized treatment further in the future. Increased knowledge of the effects of irradiation on TME cells may help in the optimization of treatments that involve RT.

## Figures and Tables

**Table 1:** Univariate Cox regression analysis.

Cancer Type	Variable	HR	95% CI	*p* Value
**CESC**	Clinical Stage	1.3751	0.9999~1.8911	0.05
	Histologic Grade	0.7737	0.4646~1.2881	0.32
**HNSC**	Clinical Stage	1.0854	0.9396~1.2537	0.26
	Histologic Grade	1.0664	0.8763~1.2975	0.52
**UCEC**	Clinical Stage	1.9276	1.6120~2.3035	<0.05
	Histologic Grade	2.7256	1.9058~3.8981	<0.05
**THCA**	Clinical Stage	2.8735	1.5947~5.1773	<0.05
	Histologic Grade	-	-	-
**BRCA**	Clinical Stage	4.0612	1.9292~8.5491	<0.05
	Histologic Grade	-	-	-
**LGG**	Clinical Stage	-	-	-
	Histologic Grade	2.7072	1.7316~4.2321	<0.05
**GBM**	Clinical Stage	-	-	-
	Histologic Grade	-	-	-

**Notes:** HR = Hazard radio, CI = Confidence interval, “-” = No information in TCGA, cannot be calculated. *p* value < 0.05 indicates statistical significance. HR = 1 indicates the variable has no impact on the outcome. HR < 1 indicates that the variable decreases the likelihood of the outcome. HR > 1 indicates that the variable increases the likelihood of the outcome.

## Supplementary Materials

The following are available online at https://www.mdpi.com/2072-6694/12/4/957/s1, Table S1: Summary of the TCGA data, Table S2: Detailed information for the 7 cancers., Table S3: Description of the 3 predictable genes, Table S4: Univariable and multivariable cox regression analysis of 3-gene signature, Figure S1–S7: Correlation matrix for 7 cancer types, Figure S8: DEGs between high immune level group and low immune level.
