# Supplementary material for: Pan-Cancer Analysis of Radiotherapy Benefits and Immune Infiltration in Multiple Human Cancers
Source: Cancers (Basel). 2020 Apr 13;12(4):957. doi: 10.3390/cancers12040957 (PMC7226004; doi:10.3390/cancers12040957)
Supplement: Supplementary file 1 [file cancers-12-00957-s001.zip › cancers-750264-supplementary/Supplementary.doc]

**Supplementary Table S1. Summary of the TCGA data.**

**Supplementary Table S2. Detailed information for the 7 cancers.**

**Supplementary Figure S1. Correlation matrix for BRCA**

**Supplementary Figure S2. Correlation matrix for CESC**

**Supplementary Figure S3. Correlation matrix for GBM**

**Supplementary Figure S4. Correlation matrix for HNSC**

**Supplementary Figure S5. Correlation matrix for LGG**

**Supplementary Figure S6. Correlation matrix for THCA**

**Supplementary Figure S7. Correlation matrix for UCEC**

**BRCA PATIENTS CHARACTRISTIC**. For BRCA patients, 98.88% patients were female, 1.12% cases were male. Tumor stage diagnosis included 199 (30.7%) cases of stage I, 610 (29.3%) of stage II, 244 (15.3%) of stage III, and 22 (24.7%) cases of stage IV. Besides, 543 patients received RT, 437 patients did not receive RT, 87 patients didn’t mentioned. Based on the ESTIMATE algorithm, stromal scores ranged from -2,164.13 to 2,050.55, and immune scores were distributed between -1,724.48 to 3,459.35, respectively.

Additionally, according to the 2019 American Cancer Society's statistics, the average 5-year survival rate for women with invasive breast cancer is 90%. This statistics result is basically consistent with our results (Figure 1D and Figure 1H). Moreover, because of the high survival rates (up to 95%) for BRCA patients in the first two years, the survival curves become intertwined and indistinguishable at this stage. In addition, a patient with a survival of more than 10 years is generally considered to be cured clinically. Therefore, we choose 557 patients who survive period of 2-10 years for further analysis. Finally, to illustrate the effect of RT and immune infiltration on the prognosis of patients, we performed four comparisons based on whether the patient received RT or the patient’s immune score. According to the analysis, we can find that: (1) For the patients who receiving RT (314 cases), high immune score patients had better survival compared with low immune score patients (Figure 1I, p = 0.0005). (2) For those who do not receive radiation therapy RT, high immune score patients also had better survival compared with low immune score patients (Figure 1J, p = 0.2735). (3) For patients with high immune score, patients receiving RT had better survival (Figure 1K, p = 0.0497). (4) For patients with low levels of immunity, no matter receiving radiotherapy or not did not affect the prognosis of patients (Figure 1L, p = 0.9291).

**Supplementary Figure. S8**. DEGs between high immune level group and low immune level. (A - B) 268 differentially expressed genes were screened, 4 of which were up-regulated and 264 were down-regulated. (C) GO enrichment analysis, Enrichment analysis showing the gene networks and enrichment of various pathways. Colors correspond to different cluster IDs. (D) Bar graph showing the top 20 results from the enrichment analysis.

**Supplementary Table S3. Description of the 3 predictable genes.**

**Supplementary Table S4. Univariable and multivariable cox regression analysis of 3-gene signature**
